# Comparative Analysis of Endodontic 0.15 Stainless-Steel K-Files: Exploring Design, Composition, and Mechanical Performance

**DOI:** 10.3390/dj12020029

**Published:** 2024-01-31

**Authors:** Abayomi Omokeji Baruwa, Filipa Chasqueira, Sofia Arantes-Oliveira, João Caramês, Duarte Marques, Jaime Portugal, Jorge N. R. Martins

**Affiliations:** 1 Faculdade de Medicina Dentária, Universidade de Lisboa, 1600-277 Lisboa, Portugal; baruwaabayomi@edu.ulisboa.pt (A.O.B.); sofiaaol@campus.ul.pt (S.A.-O.); carames@campus.ul.pt (J.C.); duarte.marques@campus.ul.pt (D.M.); jaimeportugal@edu.ulisboa.pt (J.P.); 2 Instituto Universitário Egas Moniz, 2829-511 Monte da Caparica, Portugal; achasqueira@egasmoniz.edu.pt; 3 Grupo de Investigação em Materiais Dentários (BIOMAT), Unidade de Investigação em Ciências Orais e Biomédicas (UICOB), FMDUL, 1600-277 Lisboa, Portugal; 4 LIBPhys-FCT UID/FIS/04559/2013, 1600-277 Lisboa, Portugal; 5 Instituto de Implantologia, 1070-064 Lisboa, Portugal; 6 Centro de Estudos de Medicina Dentária Baseada na Evidência (CEMDBE), 1600-277 Lisboa, Portugal; 7 Grupo de Investigação em Bioquimica e Biologia Oral (GIBBO), Unidade de Investigação em Ciências Orais e Biomédicas (UICOB), 1600-277 Lisboa, Portugal

**Keywords:** buckling, endodontics, glide path, microhardness, root canal therapy

## Abstract

To establish a glide path, smaller files (up to size 0.15) with tapers of 2% are commonly used as pathfinding files. They pre-shape the root canal space before transitioning to larger taper endodontic instruments, aiming to prevent procedural errors. This study aimed to compare the design, metal wire composition, and mechanical characteristics of seven different ISO size 15 stainless-steel hand files (K-File and C-File+). Ninety-one new stainless-steel ISO 15 K-files were mechanically tested. All files were inspected for deformations before the assessment. Dental operating microscope, scanning electron microscope (SEM), and optical microscope analyses were conducted on four randomly selected instruments from each group, and two instruments per group underwent an energy-dispersive X-ray spectroscopy (EDS) analysis. Buckling mechanical tests were performed using an Instron universal testing machine, and microhardness was assessed using a Vickers hardness tester. The statistical analysis employed the nonparametric Mood’s median test, with a significance level set at 0.05. The instrument design analysis unveiled variations in the active blade area length and the number of spirals, while maintaining consistent cross-sections and symmetrical blades. Distinct tip geometries and surface irregularities were observed. While the energy-dispersive X-ray spectroscopy confirmed similar compositions, the buckling strength and microhardness values exhibited variability across for all tested files. Notably, the Dentsply ReadySteel C-File+ recorded the highest buckling value (2.10 N), and the Dentsply ReadySteel K-File exhibited the lowest (1.00 N) (*p* < 0.05). Moreover, the Dentsply ReadySteel K-File recorded the highest microhardness value (703 HVN), while the SybronEndo Triple-Flex had the lowest (549 HVN) (*p* < 0.05). While similarities in cross-section design and metal wire composition were noted among the files, variations in the number of spirals and mechanical performance were also observed. Thus, all of these factors should be considered when selecting suitable files for an efficient root canal treatment.

## 1. Introduction

The mechanical instrumentation of the root canal space necessitates the preceding use of stainless-steel hand files to explore, establish, and maintain a glide path, a critical step for the safe application of rotary nickel titanium (NiTi) endodontic files [1,2]. Ideally, stainless-steel K-files should possess compact dimensions and exhibit robustness against torsional forces, enabling them to endure the loads encountered during apical progression [3]. To achieve an effective glide path, small files, up to size 0.15, with fixed tapers of 2%, are often considered optimal for preparing root canal space up to its terminus. Traditionally termed as pathfinding files, these instruments are employed to pre-shape the length of the root canal space prior to transitioning to larger taper endodontic instruments, effectively mitigating procedural errors [4,5,6,7]. Hence, their cutting efficiency, ease of handling, and wear resistance play a pivotal role. These attributes influence the file’s dentin removal capability, manoeuvrability, and durability across multiple uses, thereby ensuring optimal treatment outcomes [8]. However, the utilization of stainless-steel hand files presents challenges due to their limited flexibility, rendering them unsuitable for highly curved or calcified canals [9] amidst their susceptibility to fracturing [10], which can result in prolonged chairside time [2,5,11].

To surmount these challenges, manufacturers have introduced mechanized NiTi glide path files that offer established advantages over manual glide path files [9,12]. Nonetheless, many clinicians continue to favour manual glide path hand files as standard practice. This preference may be attributed to factors such as familiarity, ease of use, personal preference, and notably, their stiffness, which can be advantageous when navigating complex root canals. To address the limitations associated with stainless-steel hand files, manufacturers have undertaken various modifications encompassing alterations in file geometry and design, enhancements in rigidity, and the incorporation of carbon steel to enhance efficiency [12]. These modifications aim to optimize the performance of hand files, rendering them more flexible, durable, and proficient in managing intricate root canal anatomy.

Despite these advancements, only a limited number of studies have systematically compared and analysed the physical and mechanical properties of different brands of stainless-steel K-files [2,8,13]. Hence, the current study endeavours to bridge this gap and undertake a comprehensive comparison of the design, metal wire element composition, and mechanical performance of seven distinct ISO size 15 stainless-steel hand files, which may be considered the final file in the small glide path instrument sequence prior to full root canal mechanical preparation. The null hypothesis being tested posits that there would be no significant differences in the results of mechanical tests results among the evaluated instruments.

## 2. Materials and Methods

A total of 91 new stainless-steel 0.15 K-files, each with a length of 25 mm, were obtained from seven different sources, including SybronEndo K-Files (Sybron Endo, Orange, CA, USA), SybronEndo Triple-Flex Files (Sybron Endo, Orange, CA, USA), Mani K-Files (Mani, Tochigi, Japan), API K-Files (Nilratan Tradelink Limited, New Dehli, India), Oro K-Files (Oro Dental, New Dehli, India), Dentsply ReadySteel K-Files (Dentsply, Ballaigues, Switzerland), and the Dentsply ReadySteel C-File+ (Dentsply, Ballaigues, Switzerland). A total of 13 instruments were included in each group for mechanical testing.

### 2.1. Instrument Inspection

Prior to the mechanical assessment, a meticulous visual inspection of all instruments was conducted under a dental operating microscope (Opmi Pico, Carl Zeiss Surgical, Jena, Germany) at a magnification of 13.6×. This thorough examination was aimed to identify any major deformations, and instruments displaying such abnormalities would have been excluded from the study. However, it is noteworthy that no instruments necessitated exclusion on account of deformities, indicating the overall integrity and quality of the assessed instruments in this study.

### 2.2. Design Assessment

Four randomly selected instruments from each group were subjected to further examination under a dental operating microscope (13.6×) (Opmi Pico, Carl Zeiss Surgical, Jena, Germany) to document specific parameters, including the length of the blade’s active area and the number of spirals. Subsequently, the files were mounted on a file holder and examined using a conventional Hitachi S-2400 scanning electron microscope (SEM) (Hitachi, Tokyo, Japan). This assessment aimed to evaluate the spiral geometry (symmetrical or asymmetrical), tip design, and surface irregularities. Additionally, the instruments were embedded in acrylic, transversely sectioned using a metal cutter, and observed under a laboratory optical microscope (Meiji Saitama, Japan) with 10× magnification to determine their cross-section geometry.

### 2.3. Metal Wire Elements Assessment

Two instruments from each group were analysed using energy-dispersive X-ray spectroscopy (EDS). The analysis was conducted using a Zeiss DSM 962 scanning electron microscope (Carl Zeiss Microscopy GmbH, Munich, Germany) equipped with appropriate detectors. Before analysis, the instruments were subjected to a 2 min acetone bath to ensure surface cleanliness. The instruments were then mounted onto a sample holder and placed in the microscope chamber. The vacuum was established for 5 min, and the analysis settings included a 20-kilowatt acceleration voltage, a 3.1-ampere filament current, and a 25 mm work distance. The elemental analysis was conducted using an Inca x-act EDS detector (Oxford Instruments NanoAnalysis, Abingdon, UK) supported by images obtained by backscattered electrons. The acquisitions were made with a 45 s lifetime with an approximately 30% death time. The assessment was performed on an active instrument area of 400 µm × 400 µm. A 5-min processing time was used. The semi-quantitative elemental analysis was performed using ZAF correction and the results were analysed using The Microanalysis Suite V4.14 (Oxford Instruments NanoAnalysis, Abingdon, UK) software. The percentages of iron, chromium, and nickel were documented.

### 2.4. Mechanical Assessment

Buckling mechanical tests were conducted on an Instron universal testing machine (Instron Corporation 4502; series no H3307, Bucks, England, UK) equipped with a 1 kN load cell. The sample size determination considered outcomes from the first six tested files per group and the largest difference among the seven groups. A final sample size of 6 instruments was calculated for 80% power, an alpha of 0.05, an effect size of 1.13, and a standard deviation of 0.62 (Dentsply ReadySteel K-Files vs. Dentsply ReadySteel C-Files+). Since the other 5 groups were not included in this calculation, the final sample size was increased to 10. All files were positioned perpendicular to the floor plane with their grip locked to the universal testing machine head, and their tip pointing down and stabilized on a micro slot available in a stainless-steel testing base [13]. The test involved applying a compressive stress of 1 mm per 60 s in the axial up–down direction of the file, until a lateral displacement of 1 mm was obtained. The maximum buckling load was measured in Newtons (N).

The microhardness assessment involved testing instruments on a Vickers hardness tester (Duramin; Struers Inc., Cleveland, OH, USA). Five indentations were made on each tested instrument. The sample size determination was based on the results from the first tested instrument and the largest discrepancy between groups. For an 80% power and an alpha of 0.05, a total of 15 (3 files with 5 indentations each) were deemed necessary. Each instrument preparation adhered to the American Standards for Testing Materials (ASTM) [14]. The diamond penetrator was set to a 100 g/force (gf) pressure load for 15 s, and assessments were conducted using a 40× magnification objective [15].

Both the buckling and microhardness tests were conducted by one single operator (A.O.B.) who had access to all instrument groups and testing devices.

### 2.5. Statistical Analysis

The mechanical outcomes are presented as means and standard deviations, as well as medians and interquartile ranges. The normality of the results was assessed using the Shapiro–Wilk test. Due to the non-Gaussian distribution, a nonparametric Mood’s median test was employed for both buckling and microhardness comparisons (SPSS v.28.0.0.0 [190] for Windows; IBM SPSS Statistics, Chicago, IL, USA). The level of significance was set at 0.05.

## 3. Results

**Figure 1:**
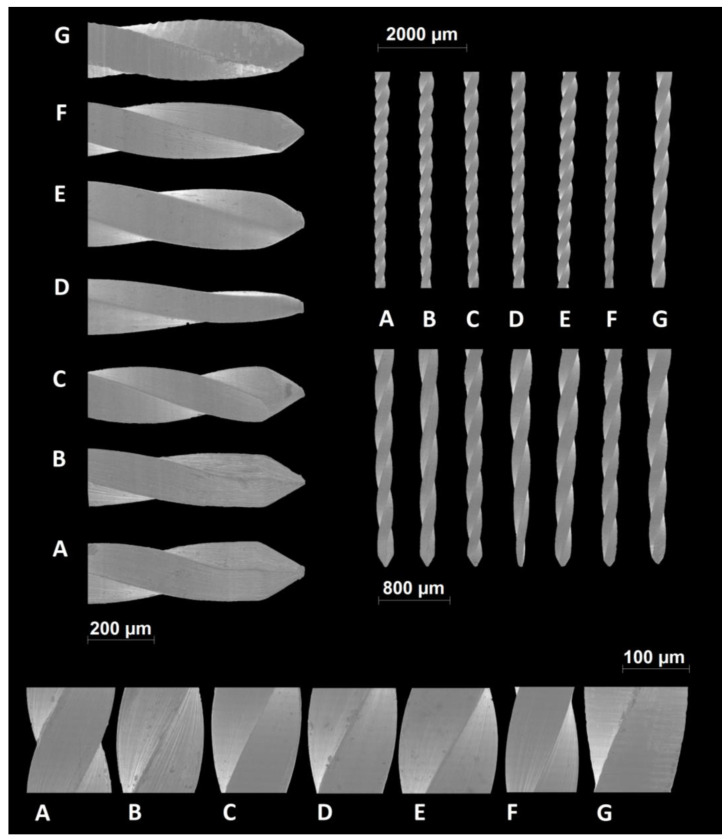
Representative images of the scanning electron microscopic analysis. Different instrument tips (**top left**) and symmetrical instrument geometries (**top right**) may be observed. On the **bottom**, the surface irregularities are documented, with SybronEndo K-Files and Triple-Flex Files showing the most irregular surfaces and Mani, API, and Oro K-Files having smoother ones ([A] SybronEndo K-Files; [B] SybronEndo Triple-Flex Files, [C] Mani K-Files, [D] API K-Files, [E] Oro K-Files, [F] Dentsply ReadySteel K-File, and [G] Dentsply ReadySteel C-File+) (n = 4).

**Figure 2:**
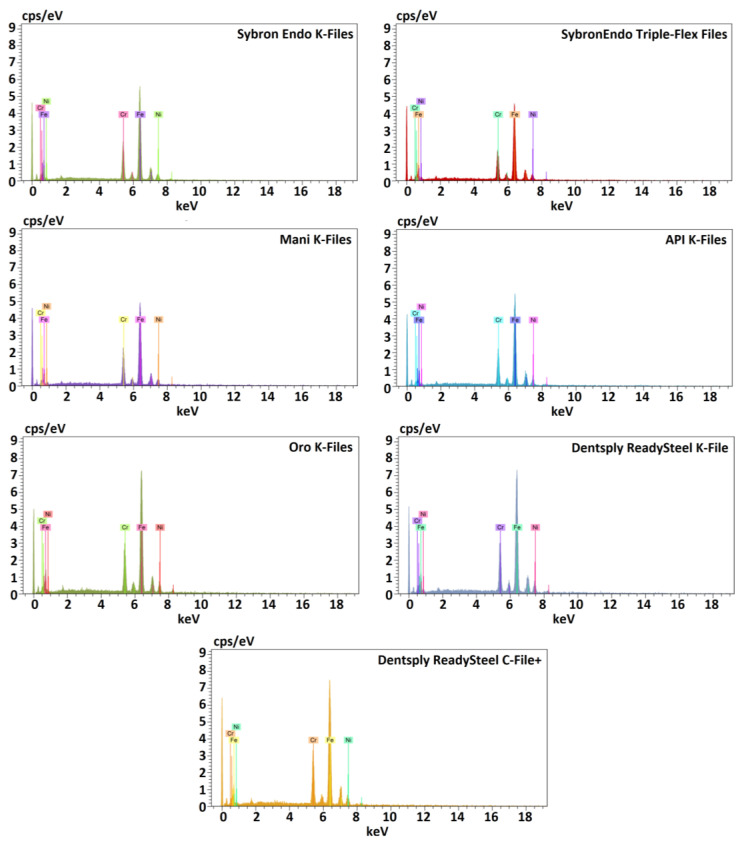
The spectrometers of the energy-dispersive X-ray spectroscopy confirmed the stainless-steel nature of all instruments’ metal wires, with equivalent element proportions between groups.

The macroscopic analysis of the instrument designs revealed distinct active area lengths on the blades, ranging from 16 mm (Dentsply ReadySteel K-File and C-File+) to 18 mm (SybronEndo Triple-Flex Files). Concurrently, the number of spirals exhibited variability, spanning from 24 (Dentsply ReadySteel C-File+) to 42 (SybronEndo K-Files). Notably, the Dentsply ReadySteel C-File+ displayed the lowest spirals/mm ratio (1.50 spirals/mm), while the SybronEndo K-Files exhibited the highest ratio (2.47 spirals/mm) (Table 1*)*. All cross-sections were characterized by square geometries (Table 1). The scanning electron microscopic analysis identified symmetrical blades across all files. Moreover, the instruments demonstrated distinct tip geometries, with the API K-Files tip appearing thinner than any other. The SybronEndo K-Files and Triple-Flex Files displayed the most irregular instrument surface, while the Mani, API, and Oro K-Files exhibited smother surfaces (Figure 1). The EDS assessment confirmed that all instruments were made of stainless-steel metal wires, with equivalent atomic percentages of iron, chromium, and nickel among the groups (Figure 2 and Table 2).

**Figure 3:**
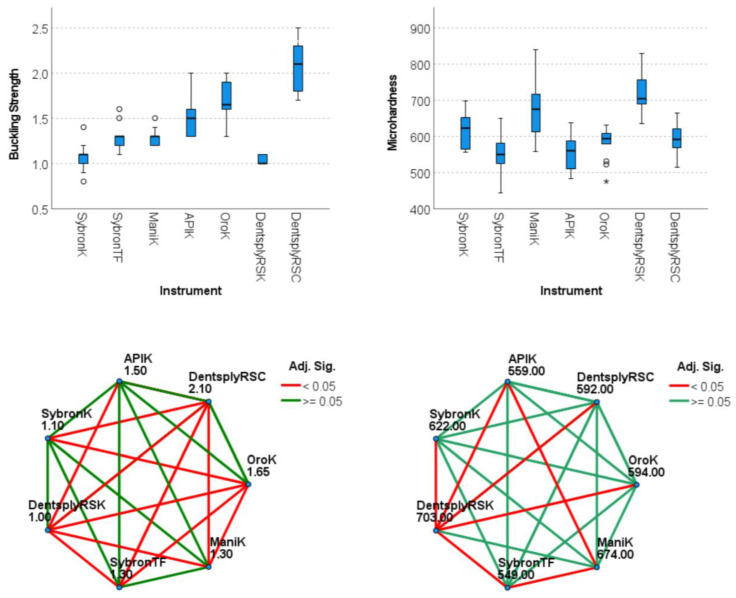
Mechanical performance for all tested instruments. The box and whisker plots (**top**) depict the buckling and microhardness results, while the pairwise comparisons (**bottom**) show the differences between groups (SybronK: SybronEndo K-Files; SybronTF: SybronEndo Triple-Flex Files; ManiK: Mani K-Files; APIK: API K-Files; OroK: Oro K-Files; DentsplyRSK: Dentsply ReadySteel K-File; and Dentsply RSC: Dentsply ReadySteel C-File+).

In terms of buckling strength, the Dentsply ReadySteel K-File and SybronEndo K-Files demonstrated the lowest strengths (1.00 N and 1.10 N, respectively), whereas the Dentsply ReadySteel C-File+ recorded the highest strength (2.10 N) (Figure 3 and Table 1). As for the microhardness, the SybronEndo Triple-Flex Files and API K-Files exhibited the lowest results (549 HVN and 559 HVN, respectively), while the Dentsply ReadySteel K-File had the highest microhardness (703 HVN) (Figure 3 and Table 1). Statistical differences were found between several files tested for both buckling strength and microhardness results (*p* < 0.05) (Figure 3).

## 4. Discussion

The main goals of the mechanical instrumentation are the removal of vital and necrotic pulp tissues from the root canal system space, thus creating enough intra-canal space to promote effective irrigation and medication and facilitate the root canal filling procedures. It should also preserve the location and integrity of the root canal apical anatomy and avoid any possible iatrogenic damage to the root and root canal morphology. It should also avoid any damage to the periapical tissues, such as the periodontal ligament or bone, while being able to preserve as much sound dentine as possible in order to allow for a good structural prognosis and strength [16]. In order to achieve these mechanical instrumentation objectives, Schilder [17], in 1974, idealized five root canal shape designs and four major biological goals. As for the root canal shaping design, Schilder advocated that the final canal shape would be (1) a continuous tapering funnel with the vertex in the apex and the base in the coronal canal opening; (2) the root canal cross-sectional diameter would be narrower at every point apically; (3) the shaped canal should preserve the original root canal morphology; (4) the apical foramen should remain in the same position; and (5) its opening remaining as small as possible. As for the major biological goals of the mechanical preparation, the author stated that (1) it should be kept confined to the root canal system only; (2) it should not force dentin debris with necrotic tissue beyond the apical foramen; (3) it should be able to remove all pulp tissues from the intra-canal space; and (4) it should create enough space for intra-canal disinfection [17].

Root canal mechanical preparation is considered as one of the most relevant steps of root canal therapy procedures [16,17]. As previously stated, it has mechanical shaping design and biological goals, of which the most important might be the physical removal of necrotic pulp tissue and to facilitate a proper root canal system space disinfection [16,17]. Although several concepts have been developed over the years, the use of endodontic files has remained mandatory, whether using manual instrumentation or mechanized canal shaping under rotation or reciprocation movements. The most frequently used manual files are the K-, H-, and K-Flex files. The K-file presents a square cross-sectional shape in small instruments and a triangular shape in larger instruments; the H-file shows a round cross-sectional design with a superior cutting efficiency when compared to K-files; and the K-Flex file has a rhomboid cross-sectional shape in order to increase the instrument flexibility. Other manual files are available on the market, such as C-, S-, Safety-H, and Hyflex files, as well as reamers or barbed broaches for pulp tissue removal, among others.

In order to achieve the root canal shape design and major biological objectives suggested by Schilder [17], the initial steps of exploring and negotiating the root canal system space, which will ultimately lead to their further enlargement and full preparation, are recommended to be performed using small patency K-files. Endodontic hand files are crucial throughout the process of mechanically shaping the root canal space during root canal treatment in achieving patency and maintaining a smooth glide path [8]. While the glide path preparation is presently undergoing a transformative shift from manual to mechanized files, the manual instrumentation relies on the tactile feedback that guides clinicians in the meticulous selection of small hand files up to a size of 0.15, ensuring the precision needed to navigate the complex root canal space. However, this manual method introduces inherent challenges, especially in curved or calcified canals, potentially leading to fatigue or iatrogenic errors [9]. Therefore, a comprehensive understanding of various aspects of these files, encompassing the design, tip configuration, cross-sectional shape, alloys used, and mechanical performance, is imperative, as they collectively influence the instruments’ overall efficiency. Such insights can aid clinicians in selecting the most appropriate hand file for specific cases, optimizing treatment outcomes. In the present study, the examined files shared a similar square cross-sectional shape and taper, with an active area spanning from 16 mm to 18 mm, and an equivalent element composition percentage. However, there were notable differences in terms of surface finishing and the number of spirals among the groups. The SybronEndo K-Files and Triple-Flex showed the highest numbers of spirals with 42 and 34, respectively, with the least surface smoothness, whilst the C-File+ (Denstply ReadySteel) displayed the fewest with 24 spirals. The number of spirals in a file plays a crucial role in its performance during root canal procedures, since more spirals enhance the flexibility but decrease the cutting efficiency and rigidity. Conversely, a lower-pitch instrument, with fewer spirals, is more effective for filing but less effective for reaming [2].

Furthermore, the files displayed distinct conical tip geometries. Previous investigations have explored the impact of tip design on canal transportation and ledging in curved canals during crown-down instrumentation [18]. The primary function of the tip of an endodontic file is to guide the file through the canal and facilitate canal enlargement. The tip design can either be active (cutting) or non-active (non-cutting), which is determined by the leading edge and the proximity of the flute to the actual tip of the file; the active cutting tip is advantageous in expediting canal enlargement and reducing the overall stress on the file [19]. The current study’s comparative analysis highlighted varied tip designs. The API K-Files featured the thinnest tip, whereas the C-File+ showcased an active pyramidal tip. Pyramidally shaped tips, as observed on the Dentsply files, have been associated with a more aggressive substrate removal in a linear fashion due to the angle and radius of their leading edge compared to files with conical or biconical tips [8]. However, this aggressiveness can lead to undesirable complications during root canal procedures such as transportation and ledging. Files with conical or biconical tips offer a more conservative approach, minimizing procedural mishaps [18]. Hence, effective navigation through complex canal anatomy and the negotiation of tight curves heavily relies on the file’s ability to maintain centricity and prevent transportation or ledging. Contrastingly, the introduction of nickel–titanium files for glide path preparation adds to the efficiency of this process, characterised by a diverse range of file sizes and motions, which minimizes the risk of procedural complications such as ledging or perforation [1,5]. This efficiency facilitates a steady and controlled progression toward the apex, preventing any deviation from the intended path. Additionally, they contribute to the prevention of fractures in subsequent enlargement instruments, further solidifying their role in enhancing procedural safety and precision [1,5,9]. However, the performance of the NiTi files is also influenced by their cross-sectional design, shape, taper, tip design, and manufacturing techniques [7]. Hence, the crucial aspect of achieving an error-free endodontic instrumentation lies in the careful selection of glide path files with an appropriate tip design and size, whether they are made of stainless steel or nickel–titanium alloys.

The present investigation assessed the endodontic instruments’ element composition using EDS tests. In order to do so, the instruments were cleaned with acetone, which is a cleaning solvent commonly used to clean metal surfaces to remove contaminants such as oil, wax, dirt, or grease, and the used settings have been recurrently used in the literature to assess metal wires’ composition [20,21]. All instruments could be classified as stainless-steel ones. The present results of the mechanical tests also demonstrated variability among instruments, prompting the rejection of the null hypothesis. The buckling strength tests indicated that the Dentsply ReadySteel K-File instruments exhibited the lowest buckling strength (1.03 N), while the Dentsply ReadySteel C-File+ showcased the highest (2.06 N). These findings could be correlated with the results on the number of spirals per millimetre. A previous study [13] showed that files presenting lower numbers of spirals tend to show higher values of buckling, which corroborates with the present report, since the C-File+ had the lowest number of spirals per millimetre of active blade (1.5 spirals/mm), while the Dentsply ReadySteel K-File was among those presenting a higher one (2.0 spiral/mm). Microhardness is indicative of a material’s resistance to deformation, tensile strength, and the cutting effectiveness of endodontic instruments [22]. In this aspect, the Dentsply ReadySteel K-File demonstrated superior performance, with the highest Vickers value of 721 HVN amongst the groups. However, it should be noticed that all groups were far above the microhardness of dentin, which is around 67 HVN [23]. This suggests that all instruments will have the ability to perform dentin cutting during root canal preparation. Additionally, and although it is not clear what the breaking point of the instruments will be, the very disparate hardness differences between dentin and the instruments also suggests that all of them will be safe to perform their work as long as they are handled correctly.

In a previous study examining the buckling and torsional resistance of 15 K exploratory hand files conducted by Kwak et al. [24], the findings revealed that the mechanical properties of instruments designed for canal exploration and glide path preparation are distinctly influenced by the file’s geometries and alloy types. The stainless-steel hand files under investigation exhibited consistent results within each group, showcasing uniformity in cross-section design and composition. However, significant variations were observed in their overall mechanical performance outcomes, emphasizing the differences in how these files respond to mechanical stress. These distinctions bear substantial implications for the selection of pathfinding files, as they directly impact critical factors such as flexibility, cutting efficiency, and the instrument’s ability to navigate complex canal anatomy. The design and composition of hand files play a pivotal role in shaping their mechanical behaviour, with elements such as the cross-sectional shape, taper, number of spirals, and tip geometry collectively contributing to the flexibility and cutting efficiency. An optimally designed hand file has the potential to offer enhanced flexibility, facilitating seamless navigation through root canal curvatures and intricacies. Additionally, this design ensures the efficient removal of dentin and debris throughout the instrumentation process. Reports have underscored the phenomenon of ongoing wear experienced by endodontic files as they interact with dentin and irrigating solutions during instrumentation [8,25]. This wear initiates progressive changes in the instruments over time, a phenomenon influenced by dentin’s inherent variability, which exhibits variations in hardness and minerals, influenced by factors such as the presence of calcification. Recognizing this dynamic nature of file wear and understanding the spectrum of dentin properties assumes an important role in endodontic practice [8]. It emphasizes the need for vigilant monitoring of instrument wear during procedures to ensure optimal efficacy and pre-empt potential complications [7]. Additionally, accounting for dentin heterogeneity allows clinicians to tailor their instrumentation techniques and select appropriate files to accommodate varying dentin conditions, ultimately enhancing the precision and success of endodontic treatments. While this study sheds light on crucial aspects, future research encompassing a broader array of instruments and tests may further enrich our understanding of the intricate interplay between endodontic hand files, dentin properties, and procedural outcomes.

In addition to understanding the intricacies of endodontic hand files, it is crucial for clinicians to account for dentin heterogeneity. Recognising the diverse nature of dentin enables practitioners to tailor their instrumentation techniques and select appropriate files that can accommodate variations in dentin conditions. This consideration enhances the precision and overall success of endodontic treatments, as different teeth may present unique challenges in terms of dentin composition and hardness. A major strength of the present study is the novel assessment of the characteristics of stainless-steel size 0.15 K-files from the point of view of their mechanical performance, geometric design, and metallurgical features. It should be highlighted that this methodology is more robust than previous stainless-steel instrument assessments, and is therefore also a methodological innovation. Although, it is essential to acknowledge the limitations of this study, which focused solely on the investigated instruments, potentially excluding other options available in the market. The variability in hand files, particularly those not included in this study, may introduce additional nuances that could impact their performance in clinical settings. Additionally, this study did not explore variables such as NiTi size 0.15 K-files (since the core of the present research was only harder instruments and, in this case, stainless steel would be the more recommended) and neglected pertinent tests, like the torsional test. Addressing these aspects and incorporating a broader range of files and tests could serve as plausible avenues for future research, providing a more comprehensive understanding of the diverse landscape of endodontic hand files and their potential applications in various clinical scenarios.

## 5. Conclusions

Despite the similarities in cross-sectional design and metal wire element composition among all tested files, variations were observed in their tip design and mechanical performance, which, regarding the latter, may be associated with design features such as the number of spirals in the active blade. Therefore, when choosing a hand file for root canal treatment, clinicians should take into consideration multiple factors to ensure successful and efficient outcomes.

## Figures and Tables

**Table 1:** Instruments’ design and mechanical performance presented as mean (standard deviation) and median [interquartile range].

Instrument	Type	Design				Mechanical Performance	
		Active Area Length	Number of Spirals	Number of Spirals/mm	Cross-Section	Buckling (N)	Microhardness (HVN)
SybronEndo K-Files	K-file	17 mm	42	2.47	Square	1.08 ± 0.16	616 ± 50
						1.10 [0.98–1.13]	622 [563–653]
SybronEndo Triple-Flex Files	K-file	18 mm	34	1.89	Square	1.31 ± 0.14	552 ± 59
						1.30 [1.20–1.35]	549 [525–587]
Mani K-Files	K-file	17 mm	34	2.00	Square	1.29 ± 0.10	670 ± 76
						1.30 [1.20–1.33]	674 [609–728]
API K-Files	K-file	17 mm	32	1.88	Square	1.54 ± 0.24	553 ± 49
						1.50 [1.30–1.68]	559 [508–592]
Oro K-Files	K-file	17 mm	32	1.88	Square	1.69 ± 0.22	582 ± 42
						1.65 [1.58–1.93]	594 [578–609]
Dentsply ReadySteel K-File	K-file	16 mm	32	2.00	Square	1.03 ± 0.05	721 ± 52
						1.00 [1.00–1.10]	703 [685–761]
Dentsply ReadySteel C-File+	C-file	16 mm	24	1.50	Square	2.06 ± 0.28	593 ± 42
						2.10 [1.78–2.30]	592 [565–630]

N: Newtons, HVN: hardness Vickers number.

**Table 2:** Instruments’ metal wire characteristics.

Instrument	Type	Metal Wire	Atomic Percentage		
			Iron	Chromium	Nickel
SybronEndo K-Files	K-file	Stainless steel	74.57	17.95	7.49
SybronEndo Triple-Flex Files	K-file	Stainless steel	74.01	17.83	8.16
Mani K-Files	K-file	Stainless steel	73.49	18.39	8.12
API K-Files	K-file	Stainless steel	74.40	17.75	7.85
Oro K-Files	K-file	Stainless steel	74.67	17.29	8.03
Dentsply ReadySteel K-File	K-file	Stainless steel	73.88	18.40	7.72
Dentsply ReadySteel C-File+	C-file	Stainless steel	74.35	18.27	7.38

## Data Availability

Data are contained within the article.
